# Impact of Angiotensin Receptor-Neprilysin Inhibitors on Patients With Acute Heart Failure Syndrome

**DOI:** 10.7759/cureus.81111

**Published:** 2025-03-24

**Authors:** David Aristizabal-Colorado, Santiago Sierra Castillo, Wilfredo Antonio Rivera Martinez, Juan Esteban Zuñiga-Terreros, Martin Ocampo-Posada, Juan David Lopez Ponce de Leon

**Affiliations:** 1 Interinstitutional Group on Internal Medicine 1 (GIMI1), Universidad Libre, Cali, COL; 2 Department of Medicine, Universidad CES, Medellín, COL; 3 Department of Endocrinology, Universidad de Antioquia, Medellín, COL; 4 Faculty of Health, Pontificia Universidad Javeriana, Cali, COL; 5 Research Group in Basic and Clinical Health Sciences, Pontificia Universidad Javeriana, Cali, COL; 6 Cardiology Service, Heart Failure and Transplant Unit, Fundación Valle del Lili, Cali, COL

**Keywords:** acute disease, heart failure, hispanic, length of stay, mortality, neprilysin

## Abstract

Introduction

Sacubitril-valsartan has shown significant efficacy in improving outcomes for certain patient populations with acute heart failure syndrome (AHFS). This study aimed to evaluate the impact of early initiation of this angiotensin receptor-neprilysin inhibitor (ARNI) therapy on in-hospital outcomes in patients enrolled in the MALEOS registry for AHFS.

Objective

This study aims to assess the impact of ARNI therapy on hospitalization duration and mortality in patients with AHFS and a left ventricular ejection fraction (LVEF) below 40% at a healthcare institution in Cali, Colombia, between 2020 and 2022.

Materials and methods

A retrospective analysis was conducted using the MALEOS registry database to identify patients with AHFS and an LVEF < 40% between January 2020 and December 2022. Patients were stratified based on whether they received ARNI therapy. Mortality and length of hospitalization were assessed using multivariate Cox regression analysis and Kaplan-Meier survival curves.

Results

One hundred and seventy-seven patients were included in this study, of whom more than 90% were Hispanic from Colombia, and 40.2% were women. In the ARNI group, 75% of patients were discharged before 12.75 days, whereas in the non-ARNI group, 75% were discharged by day 21.5. N-terminal pro-B-type natriuretic peptide (NT-ProBNP) was significantly reduced over time in the ARNI group, and mortality was lower in this group, with two fatal outcomes versus eight in the control group (Log-rank: 0.18).

Conclusions

Early initiation of ARNI therapy in patients with AHFS and reduced LVEF has significantly decreased NT-proBNP levels over time, reduced hospital stay, and improved in-hospital mortality compared to standard care. However, larger randomized controlled trials are needed to confirm these findings and assess long-term outcomes.

## Introduction

Heart failure (HF) is a prevalent cardiovascular disease affecting over 64 million people globally [1]. Primarily caused by hypertension, coronary artery disease, and cardiomyopathies, HF is a progressive condition that can lead to recurrent hospitalizations and mortality. This disease significantly impacts patients' quality of life [1]. This abnormality is evidenced by elevated levels of natriuretic peptides or by imaging or hemodynamic signs of pulmonary or systemic congestion, either at rest or induced by stress [2]. Within this complex, acute heart failure syndrome (AHFS) consists of the rapid onset of signs and symptoms of HF, with the need for treatment and occasionally hospitalization [3].

Activation of the renin-angiotensin-aldosterone system plays a central role in the pathophysiology of HF [4]. Beta-blockers, mineralocorticoid receptor antagonists (MRAs), neprilysin inhibitors, and sodium-glucose cotransporter-2 (SGLT2) inhibitors are medications designed to modulate the neurohormonal system and improve HF outcomes. These drugs reduce sympathetic nervous system activity, block aldosterone's harmful effects, inhibit the degradation of natriuretic peptides, and lower blood glucose levels. ARNI has been developed to increase vasodilatory natriuretic peptides and prevent the counter-regulatory activation of the angiotensin system. By targeting these key pathways, these medications help prevent cardiac remodeling, reduce symptoms, and improve survival in patients with HF. This therapy is highly effective in reducing the risk of death and hospitalization for HF [5,6]. The use of these drugs has been shown to reduce mortality in certain patient populations with AHFS, as demonstrated by large-scale clinical trials. However, the optimal choice of therapy should be individualized, considering factors such as renal function, blood pressure, and the patient's specific clinical presentation. Close monitoring is essential to identify and manage potential adverse effects [7].

Given the limited data on the impact of ARNI therapy in Hispanic populations, particularly in Colombia, with AHFS, this study aims to evaluate the association between early ARNI initiation in AHFS and in-hospital outcomes in patients enrolled in the MALEOS registry. We hypothesize that early ARNI therapy will be associated with reduced in-hospital mortality and shorter hospital stays [8].

## Materials and methods

A retrospective cohort study was performed using data from the MALEOS registry [8], a database of patients with HF at a healthcare institution serving a low-income population in southwestern Colombia. The Research Ethics Committee of Clínica Versalles (CEICV) issued approval GPC-CEI-0108. The committee is guided by the regulations governing research on human beings in Colombia: Resolution 8430/1993 of the Ministry of Health and Resolution 2378/2008 of the Ministry of Social Protection

**Figure 1:**
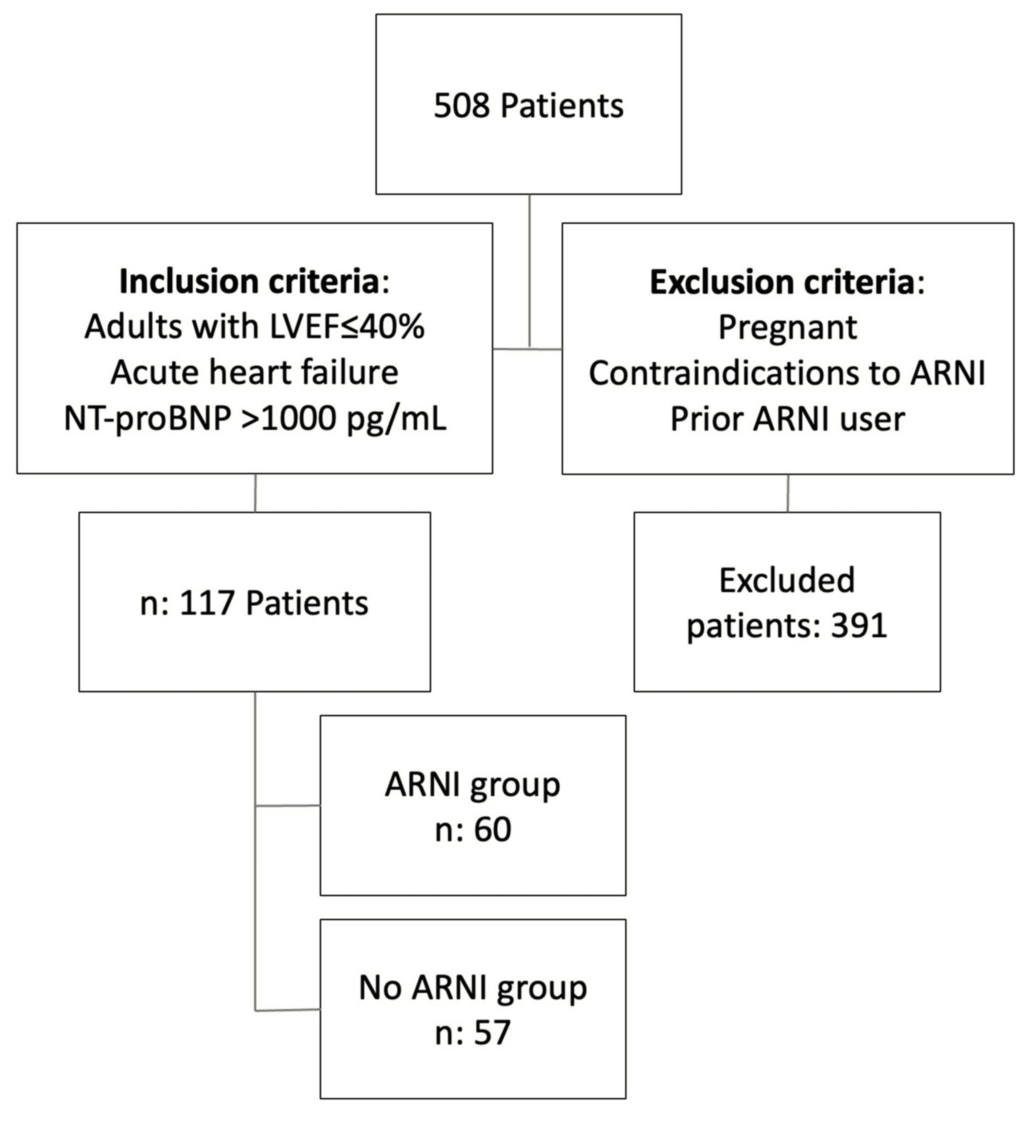
Patient inclusion diagram from the MALEOS registry ARNI: angiotensin receptor-neprilysin inhibitor, NT-ProBNP: N-terminal pro-B-type natriuretic peptide, LVEF: left ventricular ejection fraction

Patients with AHFS, a left ventricular ejection fraction (LVEF) ≤ 40%, N-terminal pro-B-type natriuretic peptide (NT-proBNP) > 1000 ng/mL, age ≥ 18 years, and hospitalization between January 2020 and December 2022 were included in the analysis. The inclusion criteria for AHFS were established based on institutional and international guidelines. Patients with contraindications to ARNI therapy, pregnant patients, prior ARNI users, or those transferred to another hospital were excluded. Data on demographics, clinical characteristics, laboratory results, treatment modalities, and in-hospital outcomes were extracted from the registry (Figure 1).

The primary outcomes of interest were in-hospital mortality, length of hospital stay, and, secondarily, the reduction in NT-proBNP levels. Data were initially cleaned using Microsoft Excel (Microsoft Corporation, Redmond, WA, USA) and subsequently analyzed with the statistical software Jamovi version 2.6.13 (The Jamovi Project, retrieved from https://www.jamovi.org), which utilizes R as its computational engine. Graphical and tabular representations were created using Power BI version 2.138.1004.0 (Microsoft Corporation, Redmond, WA, USA). Variables with more than 15% missing data were excluded, and the remaining data were imputed using dispersion measures. Descriptive statistics were used to summarize demographic and clinical characteristics. Categorical variables were compared using the Chi-square test, while continuous variables were analyzed using Student's t-test or the Mann-Whitney U test, depending on their distribution. The t-value for Jamovi was calculated using the R package.

The Kaplan-Meier method was employed to estimate survival curves, and the log-rank test was used to compare survival between groups. A multivariate Cox regression analysis was performed to identify independent predictors of in-hospital mortality, adjusting for potential confounders such as age, sex, comorbidities, and baseline renal function. Only significant variables (p < 0.05) and those considered confounding factors based on the literature were included.

## Results

Of the 508 patients with AHFS identified in the MALEOS registry, 117 met the inclusion criteria and were included in the analysis. The demographic characteristics of the study population are presented in Table 1. Most patients were male (70, 59.8%) and had a mean age of 68.9 ± 15.8 years. The mean BMI was 24.0 ± 6.5 kg/m². The majority of patients were Hispanic. There were no significant differences in demographic characteristics between patients who received ARNI therapy and those who did not.

**Table 1:** Characterization of sociodemographic, anthropometric, and comorbidities in the study population No: number, %: percentage, M: mean (standard deviation), Me: median (p25: 25th percentile; p75: 75th percentile), χ2-value: chi-square, BMI: body mass index, NYHA: New York Heart Association, HF: heart failure p-value for comparison between groups

Variables	With ARNI (60)	Without ARNI (57)	p-value	χ2-value
	n (%)	n (%)		
Gender			0.274	1.19
Male	33 (55.0)	37 (64.9)		
Female	27 (45.0)	20 (35.1)		
Age (years) M	68.9 (15.8)	70.8 (15.7)	0.443	
Weight (Kg) Me	66.5 (p25: 58.7; p75:78.0)	69 (p25: 57.0; p75:80.0)	0.983	
Height (m) Me	1.65 (p25: 1.577; p75:1.700)	1.62 (p25: 1.560; p75:1.680)	0.187	
BMI (Kg/m²) Me	24 (p25: 21.7; p75:28.2)	26 (p25: 21.0; p75:29.0)	0.539	
Race			0.278	1.18
Afro-descendant	6 (10)	10 (17.5)		
Hispanic	47 (78.3)	43 (75.4)		
Unknown	7 (11.6)	4 (7.0)		
Diabetes mellitus	25 (41.7)	23 (40.4)	0.885	0.02
Dyslipidemia	8 (13.3)	11 (19.3)	0.338	0.76
Hypertension	43 (71.7)	43 (75.4)	0.664	0.21
Hypothyroidism	8 (13.3)	15 (26.3)	0.077	3.12
Atrial fibrillation	13 (21.7)	13 (22.8)	0.882	0.02
Obesity	15 (25.0)	15 (26.3)	0.243	1.40
Chronic kidney disease	16 (26.7)	21 (36.8)	0.237	1.40
Chronic obstructive pulmonary disease	8 (13.3)	8 (14.0)	0.912	0.01
Neoplasm	4 (6.7)	0 (0.0 )	0.047	3.93
Cirrhosis	1 (1.7)	2 (3.5)	0.529	0.39
Mitral stenosis	18 (30.0)	8 (14.5)	0.310	0.79
Prosthetic valve	1 (1.7)	2 (3.6)	0.508	0.48
HF etiology			0.824	3.69
Ischemic cardiomyopathy	31 (52.5)	35 (63.6)		
Valvular cardiomyopathy	2 (3.4)	3 (5.5)		
Dilated cardiomyopathy	2 (3.4)	1 (1.8)		
Ischemic-valvular cardiomyopathy	1 (1.7)	0 (0.0)		
Hypertensive cardiomyopathy	2 (3.4)	2 (3.6)		
Atrial fibrillation cardiomyopathy	5 (8.5)	2 (3.6)		
Other cardiomyopathies	9 (15.3)	6 (10.9)		
Unknown	7 (11.9)	6 (10.9)		
Probable cause of acute decompensation			0.254	11.3
Ischemic cardiomyopathy	14 (24.1)	14 (25.0)		
Dilated cardiomyopathy	5 (8.6)	14 (25.0)		
Valvular cardiomyopathy	3 (5.2)	2 (3.6)		
Other cardiomyopathies	12 (20.7)	15 (26.8)		
Atrial fibrillation	6 (10.3)	3 (5.4)		
Hypertension	5 (8.6)	2 (3.6)		
Chronic kidney disease	2 (3.4)	2 (3.6)		
Pneumonia	2 (3.4)	0 (0.0)		
Other causes	1 (1.7)	1 (1.8)		
Unknown	8 (13.8)	3 (5.4)		
NYHA classification			0.720	2.09
I	0 (0)	1 (1.9)		
II	10 (17.2)	10 (19.2)		
III	28 (48.3)	26 (50.0)		
IV	9 (15.5)	8 (15.5)		
Unknown	11 (19.0)	7 (13.5)		
HF classification			0.111	4.40
B	50 (83.3)	45 (78.9)		
L	10 (16.7)	9 (15.8)		
C	0 (0.0)	3 (5.3)		

Regarding comorbidities and initial characteristics of patients, it was identified that 48 (73.5%) of all patients suffered from hypertension, with similar proportions in both groups (Table 2). Among the most frequent comorbidities, 41.1% of patients had diabetes mellitus, while atrial fibrillation was common in both groups; however, mitral stenosis was more frequent in patients who received ARNI management (Table 1).

**Table 2:** Characterization of variables: LVEF and blood analysis No: number, %: percentage, M: mean (standard deviation), Me: median (p25: 25th percentile; p75: 75th percentile), LVEF: left ventricular ejection fraction, PASP: pulmonary artery systolic pressure, BUN: blood urea nitrogen, GFR: glomerular filtration rate, ProBNP: pro-B-type natriuretic peptide, MCV: mean corpuscular volume p-value for comparison between groups

Variables	With ARNI (60)	Without ARNI (57)	p-value	t-value
LVEF (%) M	27.0 (7.4)	29.9 (7.5)	0.041	-2.1
PASP Me	0.0 (p25: 0.0; p75:50.0)	0.0 (p25: 0.0; p75:46.0)	0.215	-0.22
Creatinine Me	1.1 (p25: 0.9; p75:1.4)	1.3 (p25: 1.0; p75:2.1)	0.003	-2.5
BUN Me	21.5 (p25: 19.0; p75:29.2)	28 (p25: 19.0; p75:42.0)	0.122	-2.3
GFR M	60.8 (29.2)	46.8 (28.4)	0.016	2.5
Sodium M	138.0 (3.2)	136.7 (4.9)	0.085	1.7
Potassium M	4.2 (0.6)	4.3 (1.0)	0.573	-0.56
ProBNP at admission Me	7885 (p25: 3775; p75:16600)	12350 (p25: 7145; p75: 30000)	0.004	-2.6
ProBNP control M	864.6 (3660.3)	2481 (10740.5)	0.767	-1.0
Troponin Me	6.3 (p25: 0.0; p75:18.2)	2.9 (p25: 0.0; p75:30.0)	0.930	1.0
Albumin M	0.543 (1.318)	0.807 (1.405)	0.252	-0.96
Hemoglobin M	13.1 (2.0)	12.2 (2.6)	0.064	1.9
MCV Me	88.8 (p25: 85.0; p75:91.0)	84.1 (p25: 78.6; p75:89.5)	0.053	0.51
Ferritin M	30.3 (130.9)	32.4 (102.1)	0.387	-0.1
Transferrin saturation M	0.3 (1.9)	0.6 (3.1)	0.797	-0.44

The most common etiology of AHFS was ischemic heart disease, accounting for 28 (57.9%) of cases. There were no significant differences in the etiology of AHFS between the ARNI and non-ARNI groups. Most patients were classified as NYHA class III, indicating moderate symptoms of HF. According to the Stevenson classification, most patients were classified as class B, indicating AHFS without significant hemodynamic compromise.

Patients in the ARNI group had a significantly lower LVEF compared to the non-ARNI group (27% vs. 31%, p < 0.05). The mean pulmonary artery systolic pressure was higher in the ARNI group (24.3 ± 26.4 mmHg vs. 19.5 ± 21.7 mmHg, p < 0.05). Baseline NT-proBNP levels were higher in the non-ARNI group, and a decrease was seen in NT-proBNP at control and hospital discharge in the group receiving ARNI. The two groups also differed in creatinine and glomerular filtration rate (GFR) variables. Other clinical variables showed similar values ​​between the two groups (Table 2).

The most common presenting symptoms in both groups were pulmonary edema (88, 76.5%), orthopnea (85, 72.6%), and paroxysmal nocturnal dyspnea (62, 53.0%). There were no significant differences in the prevalence of these symptoms between the ARNI and non-ARNI groups. Congestive signs, such as hepatomegaly, jugular venous distention, and ascites, were less common in both groups.

Most patients in both groups received beta-blockers (56 (93.3%) in the ARNI group and 38 (82.1%) in the non-ARNI group, p < 0.05) and loop diuretics. The use of aldosterone receptor antagonists and SGLT2 inhibitors was significantly higher in the ARNI group (p < 0.05). In contrast, inotropes and calcium channel blockers were more frequent in the non-ARNI group (p < 0.05). As a decongestion strategy, patients received loop diuretic management in equal proportions, and the use of inotropes and calcium channel blockers was more frequent in the group of patients not managed with ARNI (Table 3).

**Table 3:** Established medical management No: number, %: percentage, χ2-value: chi-square, ARNI: angiotensin receptor-neprilysin inhibitor, SGLT2: sodium-glucose cotransporter-2, ACE: angiotensin-converting enzyme inhibitor, ARBs: angiotensin II receptor blockers p-value for comparison between groups

Variables	With ARNI (60)	Without ARNI (57)	p-valor	χ2-value
	n (%)	n (%)		
SGLT2 inhibitors	40 (60.7)	6 (10.5)	0.001	38.6
ACE inhibitors or ARBs	4 (6.9)	21 (36.8)	0.001	15.2
Aldosterone receptor antagonists	40 (66.7)	23 (40.4)	0.004	8.15
Parenteral or oral iron	1 (1.7)	1 (1.8)	0.041	3.99
Inotropes	4 (6.7)	7 (12.3)	0.298	1.08
Beta-blockers	56 (93.3)	38 (66.7)	0.001	13.2
Loop diuretics	46 (78.0)	43 (75.4)	0.747	0.10
Calcium channel blockers	3 (5.0)	13 (23.2)	0.004	0.08

**Figure 2:**
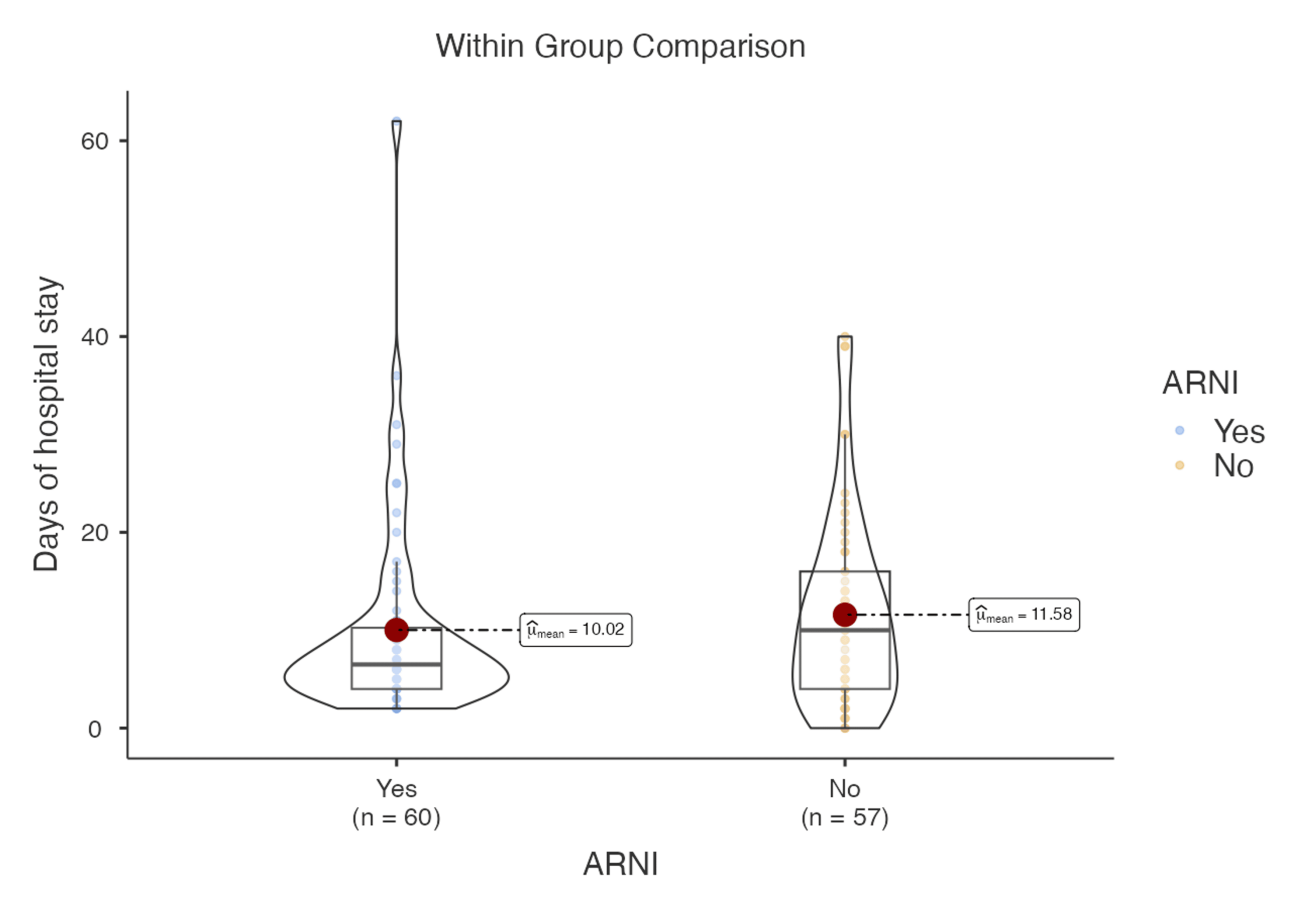
Days of hospital stay ARNI: angiotensin receptor-neprilysin inhibitor

Patients who received ARNI therapy had a shorter median hospital stay than those who did not (6.5 days vs. 9.5 days); this difference did not reach statistical significance, likely due to high variability (large standard deviation) within the data (Figure 2). This effect was more pronounced in patients with Stevenson class B HF, with a median hospital stay of 6.5 days in the ARNI group versus 9.6 days in the non-ARNI group (p = 0.08). However, this difference also did not reach statistical significance.

**Figure 3:**
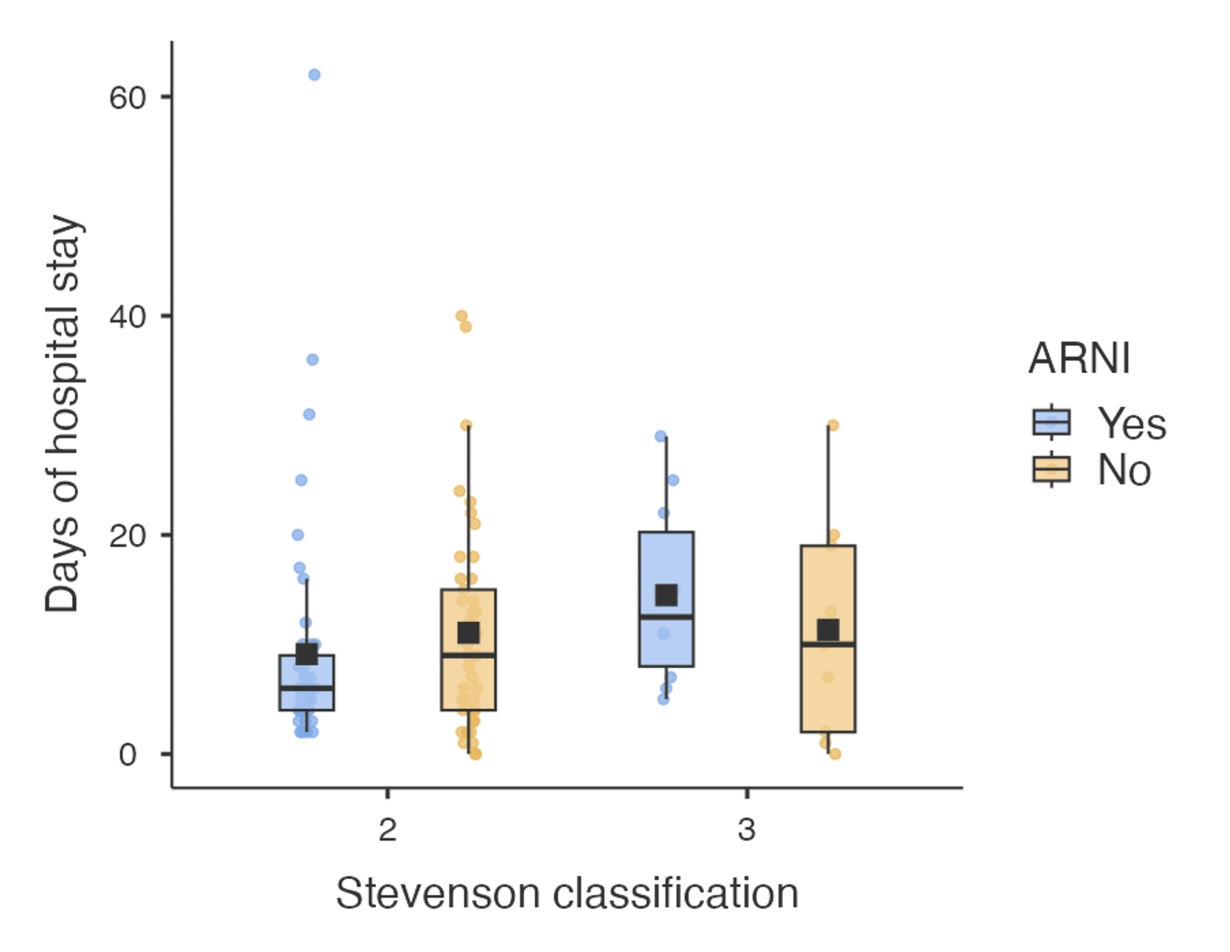
Days of hospital stay according to Stevenson classification ARNI: angiotensin receptor-neprilysin inhibitor

The survival analysis using the log-rank test revealed a difference between the groups. Still, it was insignificant in the primary endpoint of mortality (p = 0.18) for the group of patients receiving ARNI, where two patients died, compared to eight patients in the group without ARNI management (Table 4). Of the treated patient group, one death occurred in patients younger than or equal to 80 years, while the other death occurred in the group of patients older than 80 years. This resulted in a mortality rate of two (3.3%) among patients treated with ARNI. Furthermore, mortality was observed to be 0.21 times lower in patients receiving ARNI management compared to those who did not (Figure 3).

**Table 4:** Mortality in patients treated with and without ARNI No: number, %: percentage, ARNI: angiotensin receptor-neprilysin inhibitor log-rank for comparison between groups

Variables	With ARNI (60)	Without ARNI (57)	log-rank
	n (%)	n (%)	
Death	2 (3.3)	8 (14.0)	0.18
Death under 80 years old	1 (2.4)	4 (10.3)	0.39
Death over 80 years old	1 (5.5)	4 (22.2)	0.55

**Figure 4:**
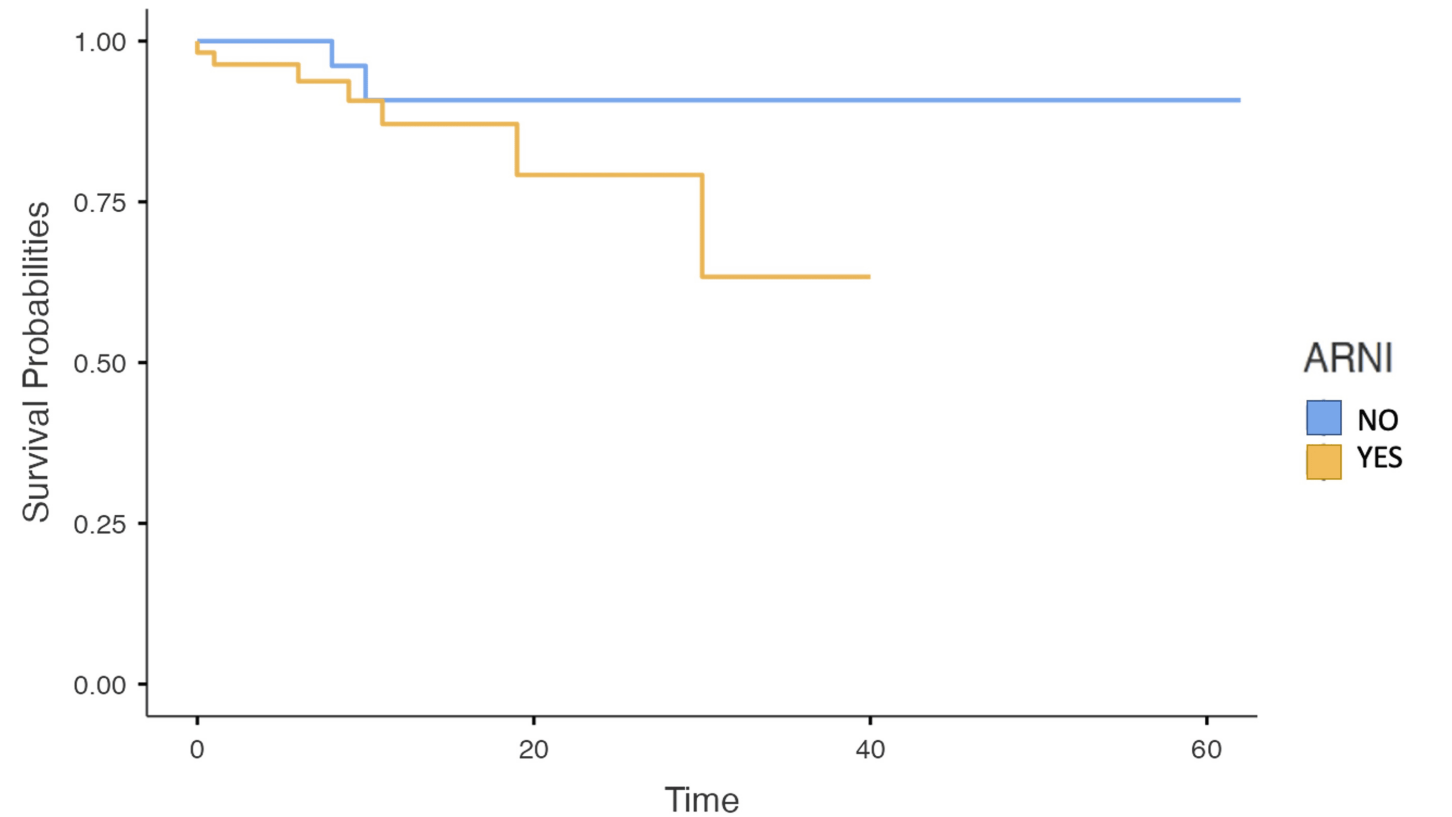
Survival curve The blue line represents patients who received ARNI, while the orange line represents the group of patients who did not. ARNI: angiotensin receptor-neprilysin inhibitor

The crude analysis showed that ARNI reduced the mortality risk (HR 0.36; 95% CI: 0.07-1.74, p = 0.204). However, this finding did not reach statistical significance. In the adjusted analysis, ARNI therapy was associated with a 31% reduction in the risk of mortality (HR 0.69, 95% CI: 0.13-3.77, p = 0.666). The wide confidence interval for the adjusted hazard ratio suggests that the estimate of the effect of ARNI on mortality is imprecise. Figure 4 represents the behavior of the mortality event in the study groups.

Variables such as age, atrial fibrillation, presence of rales, ascites, elevated blood urea nitrogen, and decreased glomerular filtration rate were associated with a higher risk of mortality in the crude model. However, in the multivariate analysis, rales were the only variable independently associated with increased mortality (HR 7.68, 95% CI: 1.43 - 41.29, p = 0.017). The presence of rales increased the mortality risk by 7.68 times compared to its absence. Although the crude analysis suggested a protective effect of ARNI therapy, this effect was attenuated after adjusting for potential confounders, such as using MRAs and beta-blockers.

## Discussion

Traditional strategies in managing AHFS have centered around three primary pillars: afterload reduction using vasodilators, preload optimization through diuretics, and contractility enhancement with inotropes [9]. Although these strategies have been the standard of care, recent studies have questioned their long-term efficacy and proposed new approaches. For instance, the GALACTIC study failed to demonstrate significant benefits from sustained vasodilation in AHFS [10]. Conversely, the PIONEER-HF study demonstrated significant clinical benefits of using ARNIs, particularly in reducing NT-proBNP levels [4]. Similarly, the STRONG-HF study highlighted the importance of early initiation and rapid titration of foundational therapy in these patients [11]. In addition to emphasizing the importance of early initiation of disease-modifying therapy, it is crucial to determine the optimal initial drug and the timing for safely initiating foundational therapy in patients with AHFS. This study underscores the early initiation of ARNI in patients hospitalized with AHFS, who were characterized by a relatively high proportion of females and individuals with ischemic heart disease. The group had an LVEF comparable to previously studied populations and experienced shorter hospital stays and lower mortality rates with ARNI therapy despite reduced use of other pharmacological treatments.

Among the characteristics of this study, a relatively high proportion of female participants was observed, accounting for 40% of the total cohort. In contrast, the PIONEER-HF study and the study by Chen et al. reported lower proportions of female participants, with 27.9% and 30.9%, respectively. Additionally, this study is notable for its predominantly Hispanic population, comprising over 75% of the participants. In contrast, the PIONEER-HF study [4] did not report this demographic, and the study by Chen et al. [12] was conducted in an Asian population [3]. The number of participants in this study is considerably smaller than that of the others mentioned, which may limit the generalizability of the findings.

Ischemic heart disease was the most frequently associated cause of HF in this study, with 56.4% of patients presenting this condition. In contrast, Chen et al. [12] reported a prevalence of 32.4%, while the PIONEER-HF study did not report the etiology of HF [4]. Notably, studies such as ADHERE have reported similar rates to those observed in this study, with 58% of patients with AHFS having ischemic heart disease [13]. On comorbidities, the PIONEER-HF study reported 72.4% of patients with hypertension, which was associated with favorable changes in NT-proBNP levels [4]. Similarly, Chen et al. found that 68% of patients with hypertension had a similar effect on NT-proBNP [12]. In our study, hypertension was the most commonly found comorbidity, affecting 73.5% of patients.

While there was no statistically significant difference in NT-proBNP changes between groups, as was seen in some of the other referenced studies, a notable difference was observed in hospital stay length. However, this difference did not reach statistical significance (p = 0.12). It suggests a potential benefit of ARNI therapy in reducing hospital length of stay. Among the groups that received or did not receive ARNI, the clinical profiles of the included patients were evaluated. It was found that 10 patients in the ARNI group and 12 in the non-ARNI group had clinical profiles of hypoperfusion (Stevenson C and L), and these patients were initiated on ARNI early after emergency room admission at the discretion of their treating physician. In these groups, the median hospital stay was 10 days (IQR 17.0) for the untreated Stevenson C group and 12.5 days (IQR 12.25) for the ARNI-treated group. In contrast, the group of clinically congestive patients (Stevenson B) showed a shorter median hospital stay for those who received ARNI. Among patients in the ARNI group, 75% were discharged within 9 days, while in the non-ARNI group, 75% were discharged by day 15, despite the ARNI group having a lower LVEF than the control group. It is important to note that the PROTECT study [14] identified in its retrospective analysis that a longer hospital stay is associated with higher readmission rates and increased 90-day mortality, consistent with results from other studies [15,16].

When evaluating in-hospital mortality, it was found that 3.3% of patients in the ARNI group experienced mortality, compared to 13.33% in the non-ARNI group. This contrasts with the mortality rate reported in MALEOS, which was 9.6% [8].

In this study, the group of patients treated with ARNI had an LVEF of 27%, compared to 31% in the group not treated with ARNI, with statistically significant differences. These findings differ from those reported in the PIONEER-HF study [4], where LVEFs of 24% and 25% were observed in the respective groups. In the study by Chen et al. [12], LVEFs of 29% and 30% were reported, with no statistically significant differences between the groups. In conclusion, unlike previous studies, the MALEOS-ARNIAHF study showed a significantly lower LVEF in the group receiving ARNI treatment. This difference can be attributed to the characteristics of the study itself, as it is retrospective, with non-randomized patients and no effort to standardize baseline characteristics for the initiation of medication.

Other pharmacologic groups were used during hospitalization, as reported in the results. Chen et al. reported that in the ARNI group, 9.4% received SGLT2 inhibitors, 69% received MRAs, and 87% received beta-blockers, whereas in the control group, 5.1% received SGLT2 inhibitors, 69.8% received MRAs, and 87.2% received beta-blockers [12]. For the PIONEER-HF study, the use of additional medications for HF was similar between the groups [4]. When evaluating the pharmacological therapies used by patients in the different studies, it is notable that foundational therapy for HF was more commonly prescribed in the ARNI group, except for beta-blockers. This difference may introduce a confounding bias, complicating the interpretation of our study's results for the ARNI group. Nonetheless, there was a trend toward reduced hospital stays and improved survival rates among patients treated with ARNI.

It is important to highlight that a recent meta-analysis suggested the significant benefits of combining neurohormonal inhibitors with newer agents like SGLT2 inhibitors and ARNI in HFrEF. This combination therapy has improved outcomes, including reduced all-cause mortality and hospitalization rates [17]. Evidence suggests that these benefits may be more pronounced in patients with more severe HFrEF, such as those in NYHA class IV. In this patient population, established therapies like SGLT2 inhibitors, angiotensin-converting enzyme inhibitors, beta-blockers, and MRAs have shown significant reductions in all-cause mortality [18].

Another potential limitation of this study is the lower baseline GFR in the non-ARNI group, which could have influenced both the initiation of ARNI therapy and patient outcomes. While we attempted to account for this factor in our analysis by including it in the model, it did not demonstrate an independent association after adjustment for other variables. However, residual confounding may still be present [19,20].

Strengths

This study provides valuable real-world data on the initiation of ARNI therapy in patients with AHFS and reduced ejection fraction, reflecting actual clinical practices. The well-characterized cohort of 117 patients allows for a detailed analysis of key clinical and biochemical parameters, including LVEF, systolic pulmonary arterial pressure, renal function, and NT-proBNP dynamics during hospitalization.

Moreover, this study addresses a Hispanic population, an underrepresented group in clinical trials, offering insights into ARNI use in this demographic. The structured comparison between patients receiving ARNI and those who did not enhance its clinical relevance, while rigorous statistical methods strengthen the reliability of the findings.

Limitations

This study evaluated patients until discharge, with no follow-up data after ARNI initiation in the outpatient setting. The sample size is smaller compared to other international studies on ARNI use. Additionally, this is a single-center, descriptive, analytical, and observational study. Although medication prescription was based on institutional and international guidelines, the study did not assess the irregular use of certain medications among patients. Moreover, the initial ARNI dose administered and its titration during hospitalization were not recorded.

Another limitation is that the study was restricted to the hospitalization period, with no follow-up after discharge, preventing the assessment of post-discharge mortality. Although SGLT2 inhibitors, MRAs, and beta-blockers in the ARNI group might have influenced the outcomes, a multivariate survival model was used to adjust for potential confounders. Furthermore, these medications are known to contribute to improved survival and a reduced length of hospital stay. Finally, a study with greater power could demonstrate statistically significant results, as observed in some previous studies.

## Conclusions

HF is a prevalent cardiovascular condition associated with recurrent hospitalizations and increased mortality. During an AHFS, the optimal choice of therapy should be individualized to improve outcomes and reduce the length of stay. In this study, patients with AHFS in the Stevenson B profile who received early ARNI treatment during hospitalization had a lower median length of stay than the control group. However, further studies with larger sample sizes and reduced confounding bias are needed to validate these findings. Regarding survival, the ARNI group showed a trend toward reduced mortality, though this result did not reach statistical significance. Additionally, rales upon emergency room admission emerged as a key predictor of mortality in the evaluated patients, highlighting its critical role in early risk stratification.
